# System-level determinants of immunization coverage disparities among health districts in Burkina Faso: a multiple case study

**DOI:** 10.1186/1472-698X-9-S1-S15

**Published:** 2009-10-14

**Authors:** Slim Haddad, Abel Bicaba, Marta Feletto, Elie Taminy, Moussa Kabore, Boubacar Ouédraogo, Gisèle Contreras, Renée Larocque, Pierre Fournier

**Affiliations:** 1 Centre de Recherche du Centre Hospitalier de l'Université de Montréal, Canada; 2 Société d'Études et de Recherche en Santé Publique, Ouagadougou, Burkina Faso

## Abstract

**Background:**

Despite rapid and tangible progress in vaccine coverage and in premature mortality rates registered in sub-Saharan Africa, inequities to access remain firmly entrenched, large pockets of low vaccination coverage persist, and coverage often varies considerably across regions, districts, and health facilities' areas of responsibility. This paper focuses on system-related factors that can explain disparities in immunization coverage among districts in Burkina Faso.

**Methods:**

A multiple-case study was conducted of six districts representative of different immunization trends and overall performance. A participative process that involved local experts and key actors led to a focus on key factors that could possibly determine the efficiency and efficacy of district vaccination services: occurrence of disease outbreaks and immunization days, overall district management performance, resources available for vaccination services, and institutional elements. The methodology, geared toward reconstructing the evolution of vaccine services performance from 2000 to 2006, is based on data from documents and from individual and group interviews in each of the six health districts. The process of interpreting results brought together the field personnel and the research team.

**Results:**

The districts that perform best are those that assemble a set of favourable conditions. However, the leadership of the district medical officer (DMO) appears to be the main conduit and the rallying point for these conditions. Typically, strong leadership that is recognized by the field teams ensures smooth operation of the vaccination services, promotes the emergence of new initiatives and offers some protection against risks related to outbreaks of epidemics or supplementary activities that can hinder routine functioning. The same is true for the ability of nurse managers and their teams to cope with new situations (epidemics, shortages of certain stocks).

**Conclusion:**

The discourse on factors that determine the performance or breakdown of local health care systems in lower and middle income countries remains largely concentrated on technocratic and financial considerations, targeting institutional reforms, availability of resources, or accessibility of health services. The leadership role of those responsible for the district, and more broadly, of those we label "the human factor", in the performance of local health care systems is mentioned only marginally. This study shows that strong and committed leadership promotes an effective mobilization of teams and creates the conditions for good performance in districts, even when they have only limited access to supports provided by external partners.

**Abstract in French:**

See the full article online for a translation of this abstract in French.

## Abstract in French

See Additional file 1 for a translation of the abstract to this article in French.

## Background

Large-scale mobilization of the international community has helped improve immunization coverage and reduce vaccine-preventable mortality [1]. Progress has been rapid and tangible [2], particularly in sub-Saharan Africa, where national programs have greatly benefited from measures to reinforce the capacity for intervention [3]. However, inequities to access remain significant, large pockets of low vaccination coverage persist, and coverage varies considerably across regions [4], districts [3], and health facilities' catchment areas [5]. In Burkina Faso, the most recent national survey of vaccination coverage showed a 41 percentage point disparity (31%-72%) between health regions with the lowest and highest complete vaccine coverage rates and a 35 percentage point disparity (58%-93%) for diphtheria, tetanus, polio and pertussis vaccine (DTPP3). Heterogeneity is also found at the district level, where coverage can vary considerably among and even within districts. There were gaps of more than 50 percentage points between the extremes of the districts in Burkina Faso and an average gap of 28 percentage points between districts within regions.

This paper focuses on district-level factors that can explain these disparities. Thus far, much less attention has been paid to district-level factors than to "micro-level" factors that might determine the propensity to have children vaccinated, either in relation to the demand side (characteristics of families, mothers and children [6]), or the supply side (characteristics of services provided locally). For example, studies in Africa, southeast Asia, and South America [7-15] have shown that immunization services' utilization is related to the acceptability, accessibility, quality, and affordability of the services provided by the health facilities and front line staff. Low vaccination coverage has been associated with lack of continuity in services (vaccine shortages, staff absenteeism, and irregularly held immunization sessions and outreach activities), poor accessibility (charges for vaccines or cards, excessive travel distance, long waiting time, and language barriers), unsuitable immunization sessions (insufficient numbers, inconvenient sessions, inappropriate schedules, and late arrival of personnel), and dissatisfaction with providers' attitudes (unfriendly behaviours, limited information transmitted to mothers, and lack of compassion/concern about the child's health).

Beyond these locally determined influences, we know little about why some districts perform better than others. Indirect evidence suggests district performance is directly related to the availability of resources required for regular supplies [15], proper functioning of the cold chain [16], and service continuity. One survey suggests that territories' vaccine coverage improves as the density of health facilities increases [17]. High turnover of senior management staff [18], restricted staff mobility [13], poor inter-sectoral collaboration [19], and faulty service organization were presumed to be related to non-performing districts. Health districts' vaccine coverage performance has also been associated with their reactions to events requiring mobilization of local capacities that could divert health workers from routine activities. These events include disease outbreaks and immunization days (IDs), about which contradictory effects have been reported [5,6,13,20,21]. Finally, health personnel motivation and attitudes [19,22] and management leadership have been identified as factors affecting the sustainability and quality of health and immunization programs [9,13,19,22,23].

Suspected managerial breakdowns in the districts are also a key focus of the Reaching Every District (RED) approach proposed by WHO to improve vaccine coverage in low-coverage areas. The RED approach targets five immunization functions: regular outreach services; supportive supervision; community links with service delivery; monitoring and use of data for action; and improved management capacities [3,24]. Others also recommend developing new strategies to improve the performance of vaccination activities [25] and training all mid-level immunization program managers in supervisory techniques and management [26].

Can performance gaps between districts and process inefficiencies at the district level be explained ultimately by outside contingencies, poor choice of intervention strategies, inappropriate organizational modalities, or suboptimal resource allocation? The answers are not clear and, to our knowledge, no systematic approaches have been undertaken to identify the determinants of these disparities. Most of the literature is based on fragmented and limited evidence and examines factors associated with vaccine coverage in only one district of a country.

This paper presents the results of a study exploring district-related factors that may account for variations in district vaccine coverage in Burkina Faso. Six districts with contrasting outcomes participated in this study. Discussions with decision makers allowed us to preselect a number of district-related factors seen as potentially influential. Based on the literature review, the research team then translated these factors into seven research hypotheses. The first four, which are focused on resources, were that, *all else being equal, immunization coverage should be higher or moving forward in districts where*:

1. donor-supported projects provide resources for routine vaccination activities;

2. the creation of new health posts has improved service accessibility;

3. health posts meet the staffing standards;

4. there is no discontinuity in supplies, nor cold chain failures.

The remaining three hypotheses refer to circumstances that are management-focused:

5. the management has introduced immunization strategies to complement the usual EPI-recommended activities;

6. the team copes appropriately with events such as outbreaks and IDs that could disrupt routine activities;

7. the District Medical Officer (DMO) demonstrates a high level of dynamism and commitment.

## Methods

### Design

The study was based on a multiple-case study design [27]. As the organizing force for all immunization activities, the district was the main unit of analysis, each district being a case. The convenience sample was intended to illustrate the diversity of evolutions in vaccination coverage. Six contrasting cases were selected: three showing increasing rates of DTPP3 and measles coverage between 2000 and 2005, two whose rates stagnated during that time frame, and one recording decreasing coverage. The cases belong to six different health regions and represent a variety of economic and socio-political conditions.

### Data collection

The cases were investigated by reviewing documents, consulting a wide array of key informants and local actors, and drawing on secondary data. Based on discussions with stakeholders and relevant literature, the research team identified potentially influential district-related factors that were subsequently reviewed and discussed with local actors. Two focus groups were held for this purpose in each district, one with the chief nurses of the primary health centres (PHCs) and another with the district medical office (DO) staff. Data related to district resources, activities, and service coverage were gathered from: i) the health information system; ii) documentary sources, such as various Ministry of Health departments' statistical reports and districts' action plans and activity reports; and iii) interviews with key informants (10-15 per district) such as DO staff, health personnel, and certain individuals who had served at the district and had since been reassigned. The indicators of vaccination coverage and the activity statistics regarding the utilization of various front line services (childbirth, antenatal visits, and curative visits) come from health statistics collated by district teams using health centres' activity registers. Documentary sources and interviews with district personnel allowed us to reconstruct the history of the presence of technical and financial partners (projects or activities financed by aid agencies or NGOs), of local vaccination strategies, or of meningitis epidemics (for further details see Table 1).

**Table 1:** Source of information and collected indicators, by independent variable.

Independent variables	Source	Indicators
Occurrence of epidemics	- Interviews with key informants	- Month and year;
		- Disease
		
Occurrence of immunization campaigns/days	- Interviews with key informants	- Month and year;
		- Antigen
		
Immunization strategies	- Interviews with key informants (DMO, person in charge of the EPI program, manager, etc.);	- % of health centers ensuring daily immunization services in fixed and outreach strategies;
	- Supervision reports and EPI report	- % of PHCs with strategies for finding drop-outs;
		- % of PHCs ensuring the management of open flasks;
		- Involvement of other actors in immunization activities (NGOs, associations, social mobilization) and the nature of their involvement
		
Profile of DMO and relationship with his team	- Interviews with DMO and key informants, manager	- Composition of the DO team (number, skills, turnover);
	- Action plan; inventory reports; supervision reports;	- Financial capacities of the DO (source and amount per year);
	- Reports from Ministry of Health	- Rate of supervisory activities integrated with report;
		- Length of DMO term of office;
		- Rate of recurrence of meetings of the DMO with the DO staff and with PHCs' chief nurses;
		- Rate of printing of DO newsletter;
		- Relation of the district action plan to the micro-plans of PHCs
		
Financial and technical partners	- Interviews with DMO or key informants, person in charge of EPI, manager	- Number of financial and technical partners in the district;
	- DO reports and action plan	- Extent of financial and in-kind aid;
		- EPI activities benefiting from partners' support;
		- Period of intervention
		
Geographic access (in km)	- Interview with DMO and key informants, person in charge of EPI,	- Number of PHCs;
	- District health statistics service	- Mean number of km to access the closest PHC;
	- DO reports and action plans	- % of population covered (served) in fixed strategy;
		- % of population served by enhanced outreach strategy
		
Incidence of PHCs meeting staffing standards	- Archives of the regional and district medical offices, action plans, and inventory reports	- % of PHCs meeting staffing standards;
	- Interviews with DMO and key informants	- % of PHCs with running COGES (management committees);
		- % of COGES that balance their budgets;
		- Degree/extent of participation of COGES in activity planning, follow-up and evaluation;
		- Nature and level of financing of EPI activities by the COGES (i.e., district's autonomy in funding its EPI activities)
		
Vaccine supplies, cold chain functionality	- Interviews with DMO and key informants, person in charge of EPI, manager, PHC's chief nurse	- Yearly budget for immunization (as for action plan, COGES and others);
	- DO reports and action plans	- Number of PHCs with functional motorbike;
		- Level/extent of equipment for cold chain;
		- Number of PHCs having experienced failures in vaccine supplies, cold chain, or means of transport and communication (month/year, length and nature of the event, number of PHCs affected)

### Analysis

The analyses used a qualitative approach based on a "pattern matching" system to compare the situation encountered against theoretical propositions derived from the hypotheses [27]. The analyses consisted of three phases. First, a qualitative time-series analysis was carried out in each case. Information from a variety of data sources was organized to highlight the temporal changes and unforeseen events observed over the dependent and independent variables' evolution. We examined each district's 2000-2005 trends in immunization coverage for DTPP3 and measles. We then compared historical trends for each independent variable with trends in DTPP3 and measles immunization coverage. In order to obtain a general overview of the evolution of the utilisation of front line services by the population, the analysis of the evolution of vaccination coverage was compared with that of antenatal consultations and of childbirths that occurred in the health facilities. The research team interpreted these trends, using mural graphs that summarized, for each case, the various data series and key events that occurred during the observation period. Simplified versions of these graphs are presented in the Results section.

In the second phase, cases were cross-analyzed, taking each hypothesis individually. This approach fed into the interpretations by means of a replication process aimed at maximizing internal validity. Rather than literal replication, we favoured theoretical replication [27] to see whether predicted patterns were found across and within cases (whether, for example, repeated events--e.g. the replacement of a DMO, followed by the replacement of that replacement--would allow us to test a hypothesis several times during the observation period).

The final phase involved returning to the field to discuss the results with the vaccination teams who participated in the group interviews. One or two field visits per district were carried out. These discussions allowed us to validate and refine our interpretation.

## Results

The first subsection below presents results from the single-case analyses. For each district (assigned fictitious names), trends in immunization coverage are compared with trends in those factors and events that are hypothesized to have influenced immunization coverage during the period of observation. Trends in the utilization rates of antenatal consultations and outpatient visits are also considered as indicators of primary health care activity. The second subsection examines the results across the six cases from the perspective of the initial hypotheses. Drawing upon focus group discussions (FGDs), the third subsection introduces the key elements and arguments pertaining to DMO leadership.

### Case studies

#### Case I: Koya district

##### Time-series analysis

**Figure 1:**
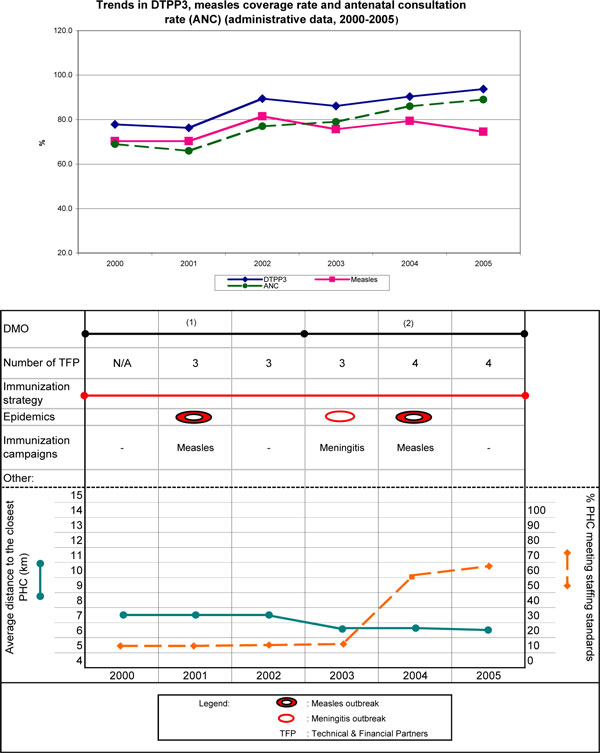
**Health district of Koya**.

In Koya, DTPP3 coverage increased from 78% (2000) to 94% (2005), maintaining immunization performances above the regional mean throughout the six-year period (with a comparative advantage of 10 to 26 percentage points). The evolution of coverage for both antigens is illustrated in the upper part of Figure 1, with their trends paralleling that of antenatal consultations. The lower part of Figure 1 illustrates historical trends and main events of the period for some key indicators accounting for the independent variables. The district experienced outbreaks of measles (2001), meningitis (2003), then measles again (2004), responding to each effectively with a campaign. The study period saw improved geographic access to the district's PHCs (from an average > 7 km to < 6 km) due to an increase in their numbers, from 23 to 30. In the first three years, only 10% to 13% of health centres met staffing standards, but this proportion increased steadily, reaching 60% in 2005. The district experienced regular disruptions in logistics, cold chain, and vaccine supplies. In 2001 and 2003, all PHCs were affected by a cold chain failure. However, the support of a few technical and financial partners (TFPs), who provided refrigerators, improved cold chain functionality. Between 2000 and 2005, two medical officers were in office, for three years each (Figure 1). Both maintained the immunization strategy introduced before 2000 that included registering foreign children in border villages and, in agricultural and farmers' valleys, attracting people at markets, in order to control targeted foreign populations.

##### FGDs - main results

In focus groups, local actors attributed Koya's consistently strong immunization performance to the district's tradition of social mobilization, going back to the EPI's establishment. Such mobilization benefits from the attendance of the nomadic populations' chiefs and the village chiefs at immunization sessions to enlist the villagers' participation, and from the support of the high commissioner and previous DMOs. Mobilization not only makes the population more responsive to immunization, but also sets the groundwork for taking immunization seriously, which helps sustain such strategies. The focus groups considered the current DMO, in office since 2003, to be less committed and apparently resting on his predecessors' laurels.

#### Case II: Bougou district

##### Time-series analysis

**Figure 2:**
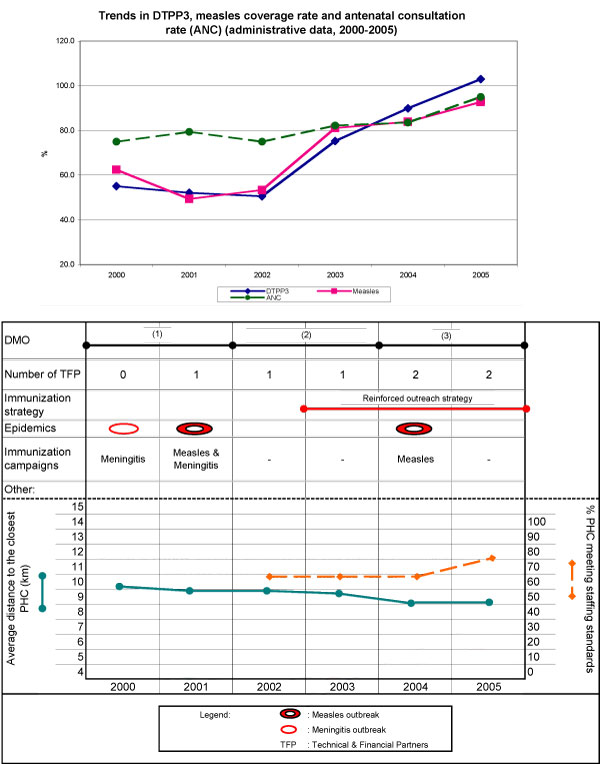
**Health district of Bougou**.

In Bougou, DTPP3 coverage decreased slightly between 2000 and 2002, with an overall uptake of 50%. Coverage increased by 25 percentage points between 2002 and 2003, and 10 per year thereafter, reaching total coverage in 2005. Measles coverage fluctuated between 50% and 60% from 2000 to 2002 and gained 30 percentage points in the following three years (Figure 2). An increasing trend is also observed in antenatal care utilization rates starting in 2002.

The district experienced several outbreaks in this period, responding in each case with a campaign: one in 2000, two in 2001, and another in 2004. Six IDs against polio were conducted in 2005 (IDs are not shown in Figures). The DMO appointed in 2002 implemented a new immunization strategy in 2003, consisting of a reinforced outreach strategy to be applied to the whole district in the first 12 months, and then subsequently only to PHCs whose immunization performance was judged insufficient or unsatisfactory. The next DMO, appointed in 2004, had been his immediate collaborator and maintained this strategy. The proportion of PHCs meeting staffing standards remained below 65% until 2004, then increased by 10 percentage points in 2005. Vaccine supply and storage were increasingly effective over the six years under observation. Before 2003, supply shortages and cold chain failures had affected several vaccines and more than 50% of PHCs. The incidence of such disruptions decreased beginning in 2003, and in 2005 only one vaccine and two PHCs were affected. While the number of PHCs experiencing a vaccine refrigerator failure did not change, the failure duration decreased by more than half, to under two weeks after 2004. TFPs in the districts have mostly contributed to logistics by donating motorbikes (28) and refrigerators (13), greatly reducing logistics failures. Geographic accessibility was comparatively poor in 2002, with an average distance of 9.5 km to the closest PHC. Two more PHCs were opened in 2003 and three in 2004, lowering the average distance to 8.7 km. The district remains nonetheless quite difficult to access, with several areas very far away from health services.

##### FGDs - main results

The increasing trend in coverage observed from 2003 onwards corresponds to the prioritization of immunization by the DO team after a situational analysis commissioned by the DMO appointed in 2002 revealed unsatisfactory EPI indicators. The resulting immunization strategy, begun in 2003, was continued by his successor. The improved performance should be ascribed to both DMOs, since both gave EPI high priority in the district's agenda and established positive relationships with the DO team and with PHC chief nurses (records show regular team meetings and regular issuing of a bulletin).

#### Case III: Mandé district

##### Time-series analysis

**Figure 3:**
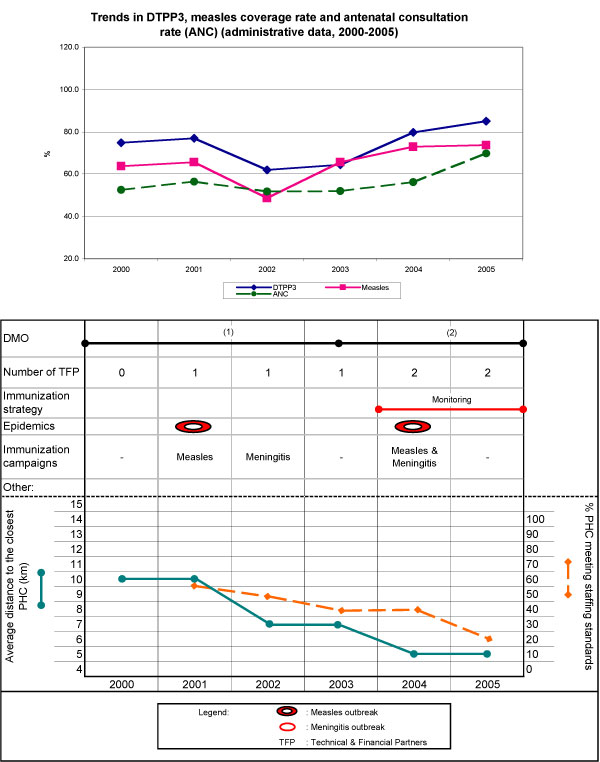
**Health district of Mandé**.

DTPP3 coverage fluctuated between 75% in 2000 and 65% in 2003 and increased steadily to 85% by 2005. Similarly, measles coverage fluctuated between 2000 and 2003 around an average of 65%, increasing to 73% in 2005 (Figure 3). The drops in both DTPP3 and measles coverage between 2001 and 2002, and the subsequent increases, are paralleled in the antenatal care utilization trend.

Mandé is characterized by relatively good geographic access, with no remote areas and comparatively low migration. The opening of three new PHCs in the six-year period halved the average distance to a health centre, from 10 to 5 km. The district experienced outbreaks in 2001 and 2004 and responded with campaigns. In each year under consideration, the district also implemented IDs; the greatest efforts were expended in 2004 and 2005, with nine and seven IDs, respectively.

Between 2002 and 2003, the PHCs' chief nurses protested (with sit-ins, strikes, activities boycotts, and local press) against the DMO, calling for his resignation on the grounds of bad management of the district's human, material, and financial resources. Many health workers also left, contributing to an already low and decreasing proportion of PHCs meeting staffing standards. In 2003, the DMO was replaced by the assistant DMO, who rapidly expanded activities and reinforced supervision of immunization activities in about half of the PHCs.

From 2000 to 2003, continuity in logistics and supplies had been very inadequate. Before 2003, less than 45% of PHCs were equipped with refrigerators and thus able to ensure the cold chain. Also before 2003, important shortages of syringes led to vaccination fees that discouraged mothers' participation in immunization sessions. In 2003, support from financial partners and a major contribution of supplies and equipment enabled more PHCs to ensure the cold chain (78% in 2004 and 91% in 2005), reestablished the supply of syringes, and supported supervision.

##### FGDs - main results

With a shift in DMO leadership, the immunization coverage trend reversed direction. Local actors decried the former DMO's lack of transparency in managing resources, his abuse of authority, and his reliance on political connections to deflect criticisms. The district's action plan was not transmitted to the team, nor were consultation meetings held with PHC chiefs or the DO team. The report of the Immunization Cluster Survey carried out in Burkina Faso in 2003 was not shared with the collaborators. The working environment had deteriorated; there were protests calling for his removal, and many workers left, thereby reducing human resources availability in the district. He was replaced in 2003 by his former assistant, who managed to restore staff confidence. She reinstated regular supervisions and consultation meetings, brought resources under better control, and significantly improved transparency in management. This led the Village Health Committee to increase its support to EPI from 4 to 10 million FCFA between 2003 and 2004, and the TFPs to allocate more resources to immunization activities.

#### Case IV: Dara district

##### Time-series analysis

**Figure 4:**
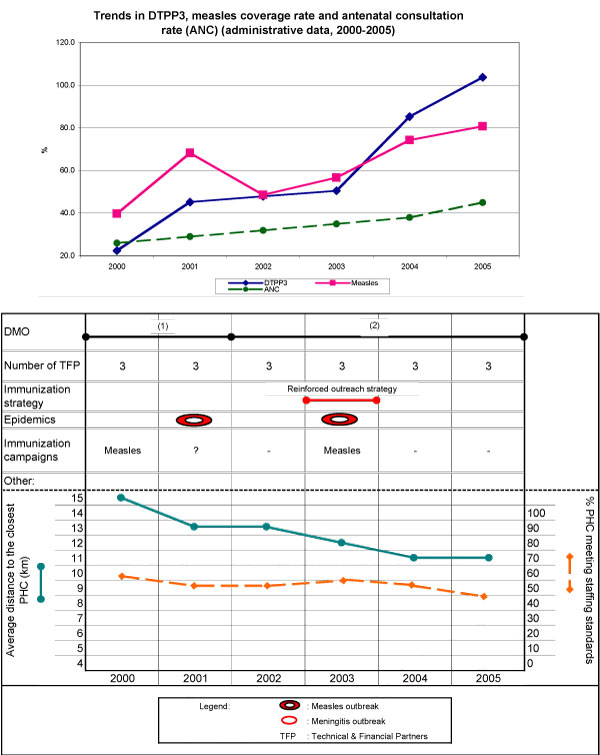
**Health district of Dara**.

In Dara, DTPP3 coverage progressed from 22% to 50% between 2000 and 2003, then jumped to 85% in 2004, and to over 100% in 2005. Measles coverage fluctuated in the first three years, then steadily increased to 81%. The district's performance remained below the regional average throughout the six-year period; however, the gap narrowed over time and immunization rates reached the regional average by 2005 (Figure 4). Utilization rates for antenatal care and outpa-tient visits also rose throughout the period, with a steeper incline starting in 2003.

In 2003, the DMO prioritized immunization, reinforcing the enhanced outreach strategy through an initiative specially geared to this district's low-density, scattered, and mobile population. Health personnel went door-to-door to identify children and vaccinate them according to their age bracket and immunization status. However, this was done only in the second semester of 2004. Financial support from foreign partners supported the initiative's implementation, as well as training in monitoring (in 2005) and logistical reinforcement (by 2005, all PHCs were equipped with motorbikes and refrigerators).

The opening of more PHCs over the six-year period improved geographic access from 15 to 12 km between 2000 and 2003, and to 11 km by 2005 (Figure 4). The proportion of PHCs meeting staffing standards decreased from 57% to 46%. Epidemics occurred in 2001 and 2003, and specific campaigns were undertaken.

##### FGDs - main results

A new DMO appointed in 2002, noting weak immunization performances, was able to mobilize the team in response. Records indicate more frequent DMO visits to PHCs after his arrival, as well as regular supervisions and statutory meetings. He developed an action plan and introduced a bonus for immunization agents.

The DMO's commitment and the resources injected into immunization starting in 2003 are reflected in an increase in coverage. While an undeniable improvement was observed, the extent of the increase is questionable. Local actors share the authors' concern about the validity of three-digit immunization numbers; however, because this district, being on the border, attracts users from outside the country, coverage rates as compiled by the information systems may indeed surpass 100%.

#### Case V: Dié district

##### Time-series analysis

**Figure 5:**
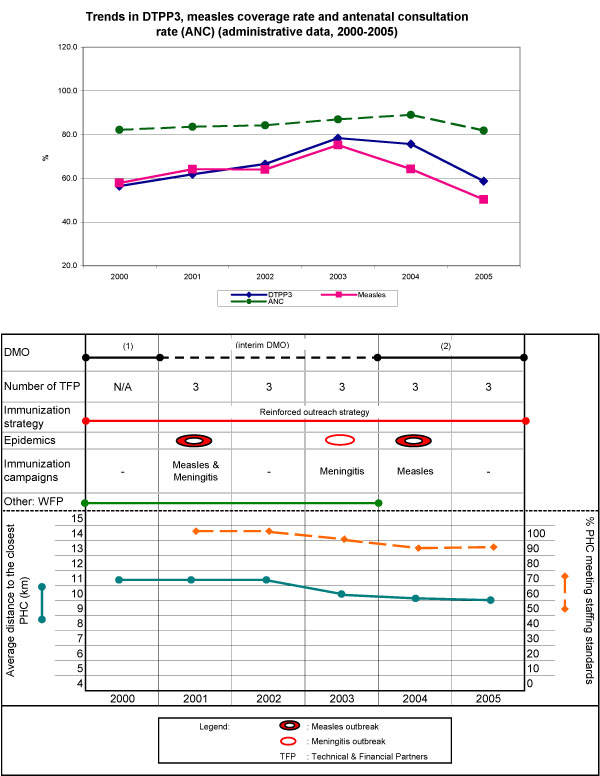
**Health district of Dié**.

Dié experienced two distinct trends in immunization coverage, with rates increasing between 2000 and 2003, then decreasing afterwards. Immunization performance has, however, been consistently below the regional average. The DTPP3 coverage rate went from 56% to 78% between 2000 and 2003, then back to 58% in 2005. The measles coverage rate rose from 58% to 75% in the first three years, and dropped back to 50% in 2005 (Figure 5). Utilization rates for antenatal care and outpatient visits also began to fall in 2004.

The district experienced four outbreaks in this six-year period: two of measles (2001, 2004) and two of meningitis (2001, 2003), each followed by campaigns. Moreover, one to three IDs targeting polio were carried out every year, then six in 2005. Increasing the number of PHCs over the six years from 22 to 28 reduced the average distance to the closest health centre from >11 km to <10 km. In the first few years, 100% of PHCs met staffing standards, but that proportion began to fall in 2003. Two of the most important disruptions in vaccine supplies occurred in 2002 and 2003. Over the six years, the district enjoyed the substantial support of several TFPs every year, three of which supported the EPI. Thus, the district's resources were significant and expanded over the years in the form of refrigerators, vaccines, equipment, and motor-bikes, to mention a few. Partners had also supported the World Food Program (WFP) in the district, but this was interrupted in 2004 due to a shortage of food supplies. During the observation period, a reinforced outreach strategy was in place, introduced by the first DMO, who served until 2001; he was succeeded by an interim officer, who was confirmed in office in 2004.

##### FGDs - main results

Participants described the second DMO as authoritarian, poor at delegating, rarely present at the DO, scarcely communicative, and not engaged with his team, who had difficulty arranging meetings with him. Since 2002, that DMO had called less than 20% of statutory meetings with his team and none with the PHCs' chief nurses, resulting in a virtual absence of discussion and consultation. Those in charge of immunization activities were not included and were confined to an operational role. After his nomination in 2004, staff dissatisfaction led to departures that reduced human resources adequacy in PHCs. Members of the DO team said they missed the previous DMO (2000-2001), who had been active and productive, assertive and appreciative (sending appreciation letters to team members who did good work). During his mandate, team members had been assigned clear tasks.

Financial partners' substantial support positively affected immunization performance; some encouraged social mobilization for immunization through a program that provided food to mothers who brought their children to be vaccinated. According to local actors, this program's interruption in 2004 had a demobilizing impact on mothers and likely contributed to the steady fall in immunization performance.

#### Case VI: Boka district

##### Time-series analysis

**Figure 6:**
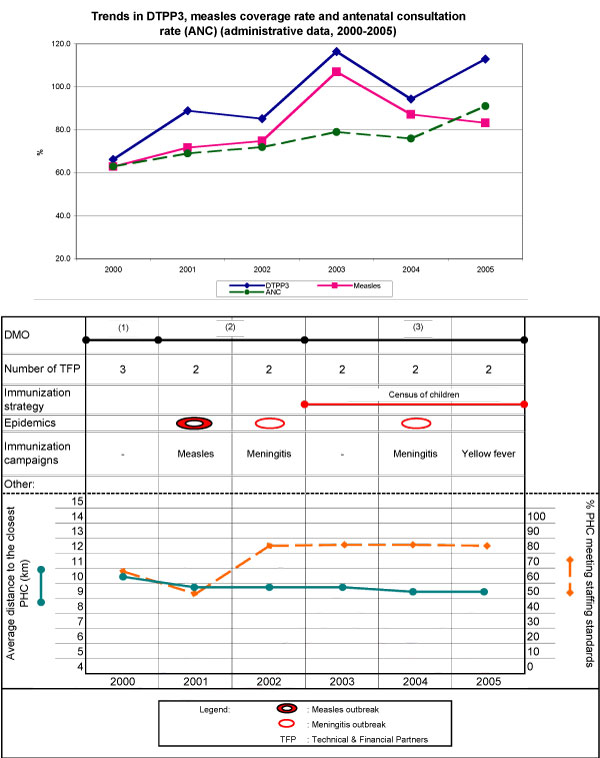
**Health district of Boka**.

Boka is in an accessible region with moderate seasonal population flows. This district registered high immunization performance over the whole period under observation, consistently over the national average (DTPP3 and measles coverage above 85% and 70%, respectively, since 2001) (Figure 6). Antenatal care and outpatient visits utilization rates were also consistently increasing.

Three DMOs served between 2000 and 2005. The last DMO, in office since 2003, introduced a census of children at the village level, continuously updated with the addition of newborns as well as incoming migrant children, in order to follow up on their immunization status. During the six years, no cold chain failure had occurred. All PHCs were equipped with motorbikes and refrigerators. The district experienced outbreaks in 2001, 2002, and 2004 and organized several IDs--eight in 2004 and 10 in 2005.

The district has enjoyed the support of partners which, although not numerous, have been quite stable in the district and have supported immunization activities.

##### FGDs - main results

In 2003 and 2004, this district attained almost complete coverage for both antigens. The district attracts clients from neighbouring districts, thus explaining the fact that coverage levels calculated on the basis of activity statistics exceed 100%. In focus groups, local actors considered the commitment and effectiveness of the latest DMO to be the determining factor. He introduced the child census and, most importantly, ensured rigorous follow-up of the enhanced outreach strategy, which included regular updating of the census register and strict adherence to the village visitation program. He also conducted four supervisions per year and regularly called PHC chiefs on his cell phone to verify the observance of activities; when a planned visit was not respected, the nurse was required to prepare a narrative report. Local actors also noted how the DMO made use of TFPs' financial support for immunization to extend services and implement new strategies such as the children census.

### Cross-case analysis

#### (1) Donor-supported projects

TFPs generally make it possible for districts to have essential resources for immunization such as vehicles and motorbikes for outreach visits, supplies, and a suitable cold chain. Some provide very specific support, as in the distribution of food in Dié which district officials consider to be a powerful inducement for women to attend vaccination sessions. However, our cross-case analyses show no unequivocal relation between the presence of a project or of external partners and district coverage performance. Even when TFPs provide significant support, performance does not appear to improve, except in districts where the DMO provides strong leadership and gives vaccination a high priority (as in Dié). Conversely, the presence of fewer TFPs does not seem to be a limiting factor in Bougou and Koya, where the EPI is a priority and DMO leadership is well established, and where everything unfolds as if the national resources (from the State, equity capital, and the Health Development Support Program) made it possible to provide adequate services. Interviews and observations in the Koya district also suggest that the TFPs' interest in funding activities in general, and those of the EPI in particular, might be related to how much collaboration there is between the local teams and foreign partners, and consequently also, to some extent, to the DMO's leadership.

#### (2) Geographic access

Several stakeholders expected the reduction in PHCs' catchment area radius to have a measurable positive impact on vaccine coverage, particularly for DTPP3, which requires several visits. The average radius is 9 km; in our sample, district radius ranged from 15 to 5 km. In Koya, where seven new centres were opened, and in Dara, where the catchment radius was substantially reduced (from 15 to 12 km), improved vaccine coverage might be explained by investment in new health facilities. However, in Dara, this increase can also be attributed to reinforced immunization strategies and the priority given to the EPI by district authorities. Prioritization by an effective leadership also explains the higher than national average immunization coverage in Boka, despite its radius being greater than the national average.

#### (3) Staffing standards

In Burkina Faso, the percentage of PHCs in a given district that meet the Ministry's staffing standard is used to estimate unmet needs. A full complement of staff enables districts to effectively implement vaccination activities such as outreach visits, react better to epidemics, and organize vaccination campaigns. This is why our respondents stressed the importance of this factor in explaining performance variations. In fact, however, our results did not support a clear association between staff levels and district performance over the years covered by the observation. In Dié we noted a steady decrease in vaccine coverage even though 90% of PHCs were fully staffed, whereas in Mandé vaccine coverage expanded while as little as 20% of PHCs had the required staff. It was the same in Koya, which, despite the low number of PHCs that met staffing norms, maintained high and steadily increasing levels of coverage between 2000 and 2003. The significantly improved human resources availability as of 2004 also had no apparent impact on vaccine coverage, which seemed to be holding steady.

#### (4) Logistics, cold chain failures

A significant improvement in logistics translates clearly, as in Mandé and Bougou, into improved vaccine coverage. In both cases, nonetheless, these improvements can also be attributed to district authorities' reinforcement of immunization strategies and TFPs' injection of resources into the EPI. Conversely, while all Koya's PHCs experienced cold chain failures in 2001 and 2003 that resulted in coverage setbacks, their amplitude was effectively contained through extended social mobilization.

#### (5) Local vaccination strategies

Beyond national immunization strategies, the vaccination performance of certain districts can be linked to their commitment to immunization and to how well they adapt their strategies to the district's context. These adaptations emerge either in response to an awareness of poor vaccine coverage that requires a specific response, or from the recognition that certain national approaches are not appropriate for the local context. They can take different forms: enhanced outreach strategies, social mobilization, monitoring or inventorying of vaccination targets. In Koya, social mobilization explains the steady, high coverage maintained despite adverse factors such as epidemics, frequent cold chain breakdowns, vaccine shortages, or staffing shortfalls. In this district, all activity indicators related to primary health care services and population coverage are at high levels, which helps to explain this sustained social mobilization. Moreover, in Bougou and Dara, the designation of the EPI as an action priority and the consequent development of new, context-adapted approaches are strongly linked with improvements in vaccination coverage. In both cases, the original idea and the implementation of these processes can be attributed to DMO leadership.

#### (6) Coping with unexpected events

##### (a) Outbreaks

At least two outbreaks of meningitis or measles were observed in each of the six districts during the period under consideration.

However, these had no measurable impact on those districts' annual vaccine coverage, and districts reacted to the outbreaks with campaigns or by intensifying vaccination activities targeting those specific diseases.

##### (b) Immunization days

Several respondents worried that IDs not only diverted the districts' attention and resources, but also demotivated populations, particularly in their "door-to-door" activities that differ from those used in routine vaccination activities, which promote encounters between health personnel and members of the population at village gathering points. They saw this as a source of confusion for mothers about where to bring their children for routine immunization in the future. Other field staff are not bothered by the IDs, for which sessions are programmed in advance and inserted into their activity programs. In practice, neither IDs nor vaccination campaigns seem to have any measurable impact on the performance of routine vaccination services, even though we counted 9 and 10 campaigns in one year in Mandé and Boka, respectively.

#### (7) DMO leadership

An important result of our participatory approach for data analysis and validation was uncovering the key role of the human factor in explaining the levels, progression, and trend reversals in districts' immunization coverage. In the two districts--Mandé, in the first years of observation, and Dié--where the DMO's presence, motivation, charisma, and collaboration were judged to be poor, there was a considerable decline not only in vaccination performance, but also, significantly, in other indicators used to monitor primary care activities. Both cases reported poorly motivated staff, low levels of collaboration between the district teams and the PHCs' chief nurses, and no strategic direction defined by the district team, nor action priorities, nor targets for available resources to be implemented in the district. Conversely, districts whose leadership was considered to be strong and of high quality, such as Boka, displayed consistently high levels of immunization coverage. In the singular case of Koya, the observed consistently high levels appear to be attributable not so much to individual leadership, but more to the collective commitment to immunization of several community actors and officials (former DMOs included). In Mandé, Dara, and Bougou, changes in leadership appeared to reverse negative trends, inducing growth in coverage and in other activity indicators.

Evidence suggests that, in Boka and Dara, the DMO promoted locally-adapted initiatives (a child census and a door-to-door strategy, respectively) in contexts characterized by large mobile populations. These two cases suggest that strong leadership helps create the conditions that facilitate expanding coverage. In both districts, a change in leadership ultimately improved the synergy with local TFPs and resulted in expanded donor-based support for the districts' action plans. This, in turn, broadened the DMOs' latitude to reinforce or expand immunization strategies.

These cases also illustrate the DMOs' human resources management skills. In Boka, the DMO was able, through rigorous supervision and follow-up, to secure his team's commitment to sustaining the child census and to ensure smooth operation in a comparatively less accessible district. In Dara, the observed extended immunization coverage reflected the DMO's ability to obtain the greatest output from a level of staffing considered largely insufficient to the district's needs. In Mandé, the intensive efforts of nine and seven ID campaigns in 2004 and 2005, respectively, were handled appropriately by the district manager with no apparent impact on routine activities. In Koya, strong social mobilization appeared to counteract the consequences of supply shortages and logistic failures; despite cold chain failures that affected all PHCs in two different years, routine functioning was not impaired.

## Discussion

Recent studies have advanced our understanding of system-related sources of disparities in coverage among countries, and of macro-level effects that are potentially attributable to human resources allocation [17], vaccine prices [6,28], decentralization [20], institutional performance [6], or aid received, whether technical or financial [2,3,28,29]. Given health districts' growing autonomy, this study is an attempt to contribute, with earlier studies [5,6,19], to better-developed factual bases to explain performance variations in vaccine coverage among districts of the same country, i.e., among territorial entities in comparable political, economic, and institutional environments. This process is all the more interesting because vaccine coverage is particularly sensitive to local health care systems' performance and constitutes a relevant marker of efficacy and good operation [15].

Four of the hypotheses refer to the potential impact of resource allocation on vaccination services efficacy and vaccine coverage progression. First, the results of our study show no unequivocal relation between the presence of a project or of external partners and the performance of districts in terms of coverage. TFPs, whether external aid organizations or cooperation projects, are currently present in nearly all the districts. However, their number, the scope of their interventions, and the types of support they provide vary considerably. Several comparative studies have demonstrated the impact on vaccine coverage of countries' access to transnational initiatives or to various forms of development aid [2,3,28,29]. Second, vaccine coverage is sensitive to district logistics, and, in particular, the cold chain. This is not surprising, given the extent to which immunization activities are contingent upon the continuous availability of the vaccines. It confirms what has been largely demonstrated [15,16], including in Burkina Faso [8]. Third, immunization coverage did not change much in districts where geographic access improved during the period of observation. As in most prevention services, there is evidence that vaccination demand is sensitive to the efforts consumers must expend to receive the services, and vaccine coverage is closely linked with geographic accessibility [7,9,10,30]. While this study does not allow us to draw clear conclusions, we nevertheless believe this should not call into question the necessity of maintaining, as a matter of common sense, strategies for developing primary health care resources in both heavily populated and relatively remote areas, as well as outreach programs to cover geographically dispersed populations. Fourth, the level of staffing in the districts' health facilities was not a key determinant of the districts' performance. The results of our study do not correspond with the expectations of decision makers and field staff, who, as mentioned, tend to consider that inadequate staffing levels are an important constraint on activities. They are also inconsistent with the results of a large comparative study [17]. However, the results did not surprise the research team, for whom it is clear that the link between human resources availability and health system effectiveness in Burkina Faso is very tenuous at both the macro and micro levels [31,32].

Two hypotheses deal with the districts' ability to cope with destabilizing situations. Seasonal epidemics and IDs habitually mobilize an important part of the districts' resources and require considerable exertion, and it has been suggested they might negatively affect routine vaccination activities. On the whole, districts seemed to adapt well and were able to adjust their vaccination activities. The seasonal epidemics did not show a tangible impact. These results tally with those of the comparative analysis of 82 countries carried out by Gauri and Khaleghian [6]. This conclusion must nevertheless be qualified because examination of the evolution of coverage at the national level shows that national performance in vaccination may be sensitive to country-wide epidemics. It may be that districts' coping capacity is limited and dependent on national directives and on the scale of the epidemics, such that they are able to adapt when faced with episodes of low or medium scale. Finally, while the study introduces factual data into the current controversy on the potentially negative impacts of IDs on routine activities, it does not resolve it. Contrary to what has been reported in India [21] and Pakistan [13], for example, neither IDs nor vaccination campaigns seem to have any measurable impact on the performance of routine vaccination services.

The core finding of our study is the primordial role of the DMO's leadership in strengthening vaccine coverage performance. Our starting hypothesis, according to which the DMO's dynamism and commitment could positively influence the overall performance of vaccination teams and services, is verified. We also found that a strong and committed leadership promotes effective mobilization of teams and creates the conditions for good district performance, even when these districts have only limited access to support from external partners.

In Burkina Faso, leadership skills are not a criterion for a DMO's appointment, nor are they fostered as a part of an institutionalized supervision of the DMO under the health region's responsibility. The choice of DMO is made at the central level without clear, standardized criteria. As a consequence, a newly appointed DMO might have little experience or technical knowledge of management, and may scarcely be interested in public health matters. Supervision and training that should be assured by the regional medical office is generally lacking. Our results suggest that leadership skills should receive more attention when a DMO appointment is considered, as well as throughout a DMO's mandate, through adequate support and supervision.

Some studies show that immunization services and, more generally, the performance of health districts are linked to the professional and ethical practices [5,19,33,34], commitment, efforts, and motivation of health personnel [13,19,22]. Deficiencies in these qualities arise largely from poor managerial skills and inadequate leadership of the health districts [35]. However, the role of the human factor in local health care system performance remains largely unexplored; it is virtually absent in the technical and administrative institutional discourse and is usually totally obscured by decision makers and development agencies [35]. Preferred strategies such as the RED approach refer to them only indirectly, either in terms of improving governance [3,24] or strengthening the management capacities of mid-level managers [26]. Even if things seem to be slowly progressing, the discourse around factors that determine the performance or breakdown of local health care systems in lower and middle income countries (LMICs) remains largely concentrated on technocratic and financial considerations, targeting institutional reforms, resource availability, or health services accessibility.

Initially, the study was not planned to be an in-depth analysis of DMO leadership and neither the specification of study variables nor the analysis could have been based on a leadership framework developed *a priori*. It is therefore difficult to ascertain precisely and measurably the leadership qualities of the different DMOs who served in the six districts during the period under consideration, and this is definitely a limitation of our study.

Empirically, from interviews with the field teams, the idea emerged that certain qualities of the DMO could play a key role in the performance of vaccination teams and services. These qualities are presented in Table 2. Given the limitations mentioned earlier, this list is provided for illustration purposes only, with no assumptions regarding its validity outside the context of this study. More in-depth studies are required to identify clearly the key elements of leadership in the context of managing district teams and to document the impacts, still not well understood, of the human factor on district performance.

**Table 2:** Elements of leadership that could affect the performance of vaccination teams and services.

Qualities most often mentioned by field staff during focus groups were:
(1) Exercising authority:"leadership" authority, personality, charisma, ability "to keep on top of things"
(2) Managing teams: taking care to transmit and share information, listening, holding regular meetings, motivating staff, encouraging staff participation in decision-making
(3) Ability to create a good working environment
(4) Professionalism, voluntarism: able to analyze situations; volunteering; able to innovate and look for new solutions; undertaking new approaches; responding well to unanticipated situations; able to have his decisions recognized at the central and regional levels
(5) Diligence: being always present in the district
(6) Transparency in the management of resources

Because of the relatively exploratory character of our approach and its setting in the reality of Burkina Faso, one limitation of the study is the extent to which the results may be generalized. Also, the local context and the participative process led us to concentrate on a relatively limited number of exogenous and endogenous factors to explain differences observed in the degree and progression of coverage in only six districts. Large-scale studies might make it possible to explore further the different mechanisms of causality and the means by which external environments, the human factor, available resources, and institutional elements determine the efficiency and efficacy of district vaccination services.

## Conclusion

The key to success appears to reside in the districts' ability to assemble a set of favourable conditions in which the human factor might play a major role. But the importance of leadership should not overshadow the fact that bringing together these favourable conditions and, particularly, implementing initiatives that are district-specific and adapted to their realities, such as the enhanced outreach strategy or child census, require at least a minimal amount of financial and technical resources. Our results indicate that a district can get these resources either from redirecting the funding priorities for its action plan and reallocating its own resources (supplied by the central authorities), or else from TFPs. Our observations suggest, in particular, that a change of team and new district leadership could, as was recently seen in The Gambia, provide the impulse needed to create a more collaborative dynamic with local TFPs [35] and encourage them to provide even greater support to the district's action plans.

Two decades ago, when the primary health care model was becoming widely adopted, Simmonds [22] advocated for devoting more substantial efforts to strengthening leadership capacities and setting up appropriate incentive systems. These recommendations, too often ignored in the implementation of decentralization, remain very relevant today.

## List of abbreviations used

DMO: District medical officer; DO: District medical office; RED: Reaching Every District; PHC: Primary health centres; DTPP3: Diphtheria, tetanus, polio and pertussis vaccine; FGDs: Focus group discussions; ID: Immunization day; TFPs: Technical and financial partners; WFP: World Food Program; EPI: Expanded Program on Immunization; LMICs: Lower and middle income countries.

## Competing interests

The authors declare they have no competing interests.

## Authors' contributions

SH and AB are the principal co-authors and contributed equally to this work. They took part in every phase of the study and, as principal investigators, are responsible for the scientific aspects of this article. All the authors were involved in the preparation of the research project, the analyses, and the drafting of the article. MK and ET were responsible for relations with decisions makers and stakeholders. MF supervised the data analysis and the formulation of results. GC contributed to the literature review and data analysis. PF provided scientific support throughout the project. All authors provided feedback on, and made revisions to the manuscript.

## Supplementary Material

Additional file 1 Abstract in French. Click here for file
