# Supplementary material for: System-level determinants of immunization coverage disparities among health districts in Burkina Faso: a multiple case study
Source: BMC Int Health Hum Rights. 2009 Oct 14;9(Suppl 1):S15. doi: 10.1186/1472-698X-9-S1-S15 (PMC3226234; doi:10.1186/1472-698X-9-S1-S15)
Supplement: Additional file 1 — Abstract in French. [file 1472-698X-9-S1-S15-S1.pdf]

# Déterminants systémiques des disparités de couverture vaccinale entre districts sanitaires du Burkina Faso : une étude de cas multiple.

Slim Haddad, Abel Bicaba, Marta Feletto, Elie Taminy, Moussa Kabore, Boubacar Ouedraogo, Gisèle Contreras, Pierre Fournier

## Résumé

### Problématique

Malgré les progrès rapides et tangibles dans la couverture vaccinale et la mortalité évitable par la vaccination enregistrés en Afrique subsaharienne, de larges poches de faible couverture vaccinale persistent et les iniquités d'accès à la vaccination demeurent tangibles. De fortes disparités persistent entre régions, entre districts, et au sein de mêmes districts entre les aires de responsabilités des formations sanitaires. Cet article s'intéresse aux facteurs propres aux systèmes de santé qui, au Burkina Faso, expliquent les disparités de couverture entre districts.

### Méthodes

L'étude repose sur une étude de cas multiple réalisée dans six districts présentant des niveaux de performance et des profils d'évolution de la couverture vaccinale différents. Des études préparatoires et un processus participatif associant des informateurs-clé ont conduit à l'identification d'un ensemble de facteurs systémiques susceptibles d'expliquer l'efficacité des activités de vaccination dans les districts : la survenue d'épidémies et la conduite de journées nationales de vaccination, la performance du management, les ressources disponibles pour la réalisation des activités de vaccination, et des composantes institutionnelles. La méthodologie, axée vers la reconstruction de l'évolution de la

performance des services de vaccination de 2000 à 2006, est basée sur divers documents et des entrevues individuelles et de groupe dans chacun des six districts. Le processus d'analyse des données a inclus l'équipe de recherche ainsi que les équipes de terrain.

**Résultats**

Les résultats suggèrent que les districts les plus performants sont ceux qui réunissent un ensemble de conditions favorables. Mais le leadership du Médecin chef de District (MCD) semble constituer la porte d'entrée principale et le facteur rassembleur de ces conditions favorables. Typiquement, un leadership fort et reconnu par les équipes de terrain assure le bon fonctionnement des services de vaccination, favorise l'émergence d'initiatives nouvelles en faveur de la vaccination et les protège, en quelque sorte, contre les risques inhérents à la survenue d'épidémies ou d'activités surajoutées pouvant nuire à la conduite des activités de routine. Il en est de même pour la capacité des infirmiers chefs de poste et leur équipe à s'adapter aux situations nouvelles (épidémies, ruptures de certains stocks).

**Conclusions**

Le discours sur les facteurs déterminants de la performance ou de la défaillance des systèmes locaux de santé dans les pays à faibles et moyens revenus demeure largement focalisé sur des considérations technocratiques et financières, ciblant les réformes institutionnelles, la disponibilité des ressources, ou l'accessibilité des services de santé. À l'opposé, le rôle du leadership des responsables de district et plus généralement, de ce qu'on appelle « le facteur humain », dans la performance des systèmes de santé locaux n'est évoqué que très marginalement. Cette étude montre qu'un leadership fort et engagé

favorise une mobilisation efficace des équipes et crée les conditions d’une bonne performance des districts, y compris lorsque ces derniers n’ont qu’un accès limité à des soutiens provenant de partenaires extérieurs.
